# Glia and zinc in ageing and Alzheimer’s disease: a mechanism for cognitive decline?

**DOI:** 10.3389/fnagi.2014.00137

**Published:** 2014-06-25

**Authors:** Sara M. Hancock, David I. Finkelstein, Paul A. Adlard

**Affiliations:** ^1^Synaptic Neurobiology Laboratory, Florey Institute of Neuroscience and Mental HealthParkville, VIC, Australia; ^2^Parkinson’s Disease Laboratory, Florey Institute of Neuroscience and Mental HealthParkville, VIC, Australia

**Keywords:** ageing (aging), microglia, zinc, Alzheimer disease, synapse regulation, astrocyte-neuron interactions

## Abstract

Normal ageing is characterized by cognitive decline across a range of neurological functions, which are further impaired in Alzheimer’s disease (AD). Recently, alterations in zinc (Zn) concentrations, particularly at the synapse, have emerged as a potential mechanism underlying the cognitive changes that occur in both ageing and AD. Zn is now accepted as a potent neuromodulator, affecting a variety of signaling pathways at the synapse that are critical to normal cognition. While the focus has principally been on the neuron: Zn interaction, there is a growing literature suggesting that glia may also play a modulatory role in maintaining both Zn ion homeostasis and the normal function of the synapse. Indeed, zinc transporters (ZnT’s) have been demonstrated in glial cells where Zn has also been shown to have a role in signaling. Furthermore, there is increasing evidence that the pathogenesis of AD critically involves glial cells (such as astrocytes), which have been reported to contribute to amyloid-beta (Aβ) neurotoxicity. This review discusses the current evidence supporting a complex interplay of glia, Zn dyshomeostasis and synaptic function in ageing and AD.

## Introduction

Ageing is an inevitable biological process wherein physical and mental capabilities are diminished over time, often resulting from a variety of factors such as cumulative oxidative stress and altered cell metabolism. This functional decline then ultimately results in a loss of synaptic plasticity. Ageing in itself does not require a treatment *per se*, but maintaining cognitive function into old age is a concept many aspire to. Currently, normal ageing is considered to be associated with an overall decline in cognition occurring via structural and functional brain changes over a period of time (Meunier et al., 2014). While we have a strong understanding of the physical decline that occurs in peripheral organs and systems (e.g., muscle and bone); the particular molecular and cellular changes that occur within the brain and which ultimately underlie the progression of normal ageing are yet to be fully determined. Despite the lack of consensus on the precise neural alterations that occur, it is clear that there is a fine line between healthy and pathological ageing.

## Healthy ageing vs. Alzheimer’s disease

Currently, the mechanisms underlying ageing within the brain remain poorly understood, and indeed one of the hallmarks of ageing is its variability (Meunier et al., 2014), with the preservation or loss of cognitive functions differing between individuals. The functional memory decline that does occur, however, is actually well characterized, with executive functioning, processing speed and reasoning ability declining from middle age (Deary et al., 2009). While the molecular and cellular mechanisms underlying this are yet to be fully elucidated, it is important to note the potential intersection with pathological ageing, as seen in conditions such as Alzheimer’s disease (AD). Ageing is the greatest risk factor for the development of AD, which is the most common form of age-related dementia (Mosconi et al., 2010; Reitz et al., 2011), and it has been suggested that AD may simply be an acceleration of the normal ageing process. Indeed, many of the cognitive impairments seen in normal ageing are further exacerbated in AD. Symptomatically, AD is characterized by marked deficiencies in episodic memory, attention, perception and speech (Mesulam, 1999) as well as altered mood (Lopez et al., 2001). Pathologically it is defined by the accumulation of intracellular neurofibrillary tangles (comprised of abnormally phosphorylated tau protein) and extracellular plaques (comprised of misfolded forms of the amyloid-β (Aβ) peptide) within the brain. With regards to the potential for a mechanistic overlap between ageing and AD, recent evidence points to zinc (Zn) homeostasis as key player in both normal and pathological ageing. Specifically, it has been demonstrated that there is a modulation in brain Zn homeostasis in both ageing and AD (Religa et al., 2006; Haase and Rink, 2009) that results in a neuronal Zn deficiency that may ultimately underlie the onset and progression of cognitive deficits seen in both.

## Zinc

**Figure 1:**
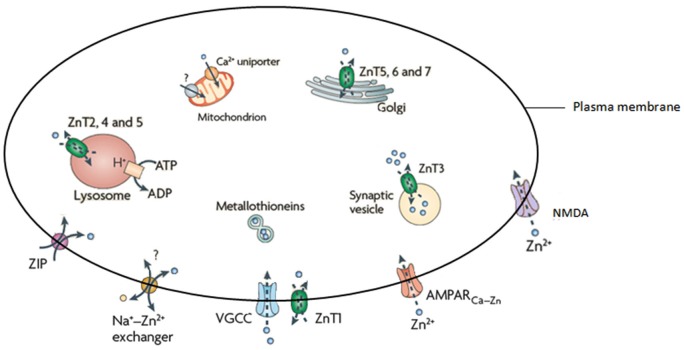
**Illustration of the proteins responsible for the homeostasis of Zn.** Adapted from Sensi et al. (2009).

Zn; an essential trace element and second in abundance in mammalian tissues (Wang et al., 2005; Paoletti et al., 2009), is critical for immunity, growth and development (Nolte et al., 2004), is a cofactor for more than 300 enzymes and is essential for the correct functioning of over 2000 transcription factors (Takeda, 2000; Levenson and Tassabehji, 2007; Jeong and Eide, 2013). The brain has the largest Zn content (Vasto et al., 2008), the levels of which are tightly controlled by three main families of proteins that have a distinct tissue and cell level pattern of localization and expression (Hennigar and Kelleher, 2012). These are; the metallothioneins (MT’s; that also coordinate a variety of other metal ions), zinc transporters (ZnT’s) and Zn-regulated and iron-regulated transporter proteins (ZIP’s; recent evidence has also implicated the presenilin family as capable of influencing Zn concentrations (Greenough et al., 2013)). Currently there are four MT isoforms, 10 ZnT’s, 15 ZIP’s and two presenilins. The ZnT’s coordinate intracellular Zn homeostasis while the ZIP’s primary function is to regulate Zn uptake (Guerinot, 2000). The role of the MT’s is to control cytosolic concentrations through the binding and distribution of Zn (Mocchegiani et al., 2001). A number of studies have examined the effect of altered MT on brain metal levels, with mice deficient in both MT-I/II (Manso et al., 2011) and MT-III (Erickson et al., 1997), for example, shown to have altered brain Zn levels. Cumulatively, these proteins are responsible for the influx and efflux of Zn^2+^ in a variety of cellular compartments, including vesicles, Golgi apparatus, and mitochondria (Figure 1).

As Zn is essential for normal brain function, Zn dyshomeostasis has been linked to a range of neurological abnormalities including (but not limited to) depression and schizophrenia (Levenson and Tassabehji, 2007), AD, amyotrophic lateral sclerosis (ALS), Down’s syndrome, multiple sclerosis (Grabrucker et al., 2011) and normal age-related cognitive decline (Adlard et al., 2014). Neurological events such as ischemia, seizures and traumatic brain injury have also been linked to altered Zn levels (Nolte et al., 2004; Grabrucker et al., 2011). In addition to this, excess Zn has been implicated in the processes that lead to cellular damage through excitotoxicity or oxidative stress (Morris and Levenson, 2012). This is particularly pertinent at the synapse where the presence or absence of Zn can be crucial.

## Zinc at the synapse (neuron-neuron)

Though it is well-known that Zn is tightly bound to macromolecules in the brain, a small number of Zn ions (approx. 10–15% total brain Zn) exist as chelatable Zn (Levenson and Tassabehji, 2007) primarily within synaptic vesicles at glutamatergic synapses (Paoletti et al., 2009; Sensi et al., 2009; Karol et al., 2010). During neuronal activation Zn is released into the synaptic cleft alongside glutamate (Lee et al., 2012) where it interacts with synaptic receptors, ion channels and transporters to modulate synaptic activity (Takeda and Tamano, 2012). Most importantly, this Zn acts on neuronal receptors and voltage-gated calcium channels (VGCC) to regulate downstream signaling pathways and neuronal processes such as; normal neuronal firing, long-term potentiation (LTP) and long-term depression (LTD) by acting on N-methyl-D-aspartate (NMDA) and α-amino-3-hydroxy-5-methyl-4-isoxazolepropionic acid (AMPA) receptors, particularly in the hippocampus. This is further evidenced by the age-dependent cognitive phenotype recently reported in zinc transporter 3 (ZnT3) knockout mice (Adlard et al., 2010), which lack synaptic Zn at the glutamatergic synapse. Interestingly ZnT3 mRNA and protein levels are decreased in AD (Beyer et al., 2009; Adlard et al., 2010). Research has also demonstrated that Zn is capable of inducing functional and conformational changes in NMDA receptors (Sirrieh et al., 2013) and is therefore considered essential for the modulation of synaptic neurotransmission (Roberts et al., 2012).

Likewise, Salazar et al. (2005) further demonstrated an intimate connection between Zn and vesicular glutamate transporter 1 (VGlut1) which is expressed in synaptic terminals that co-release Zn and glutamate. Glutamate, the major excitatory neurotransmitter in the central nervous system (CNS), is essential to synaptic plasticity underlying cognition and memory. Vglut1 and ZnT3 coexist in nerve terminals and are also co-targeted to the same vesicle population and are reciprocally regulated; Zn uptake is increased by Vglut1 expression while glutamate uptake is increased by production of ZnT3. This becomes exceedingly relevant as evidence is emerging for a role of astrocytes in Zn homeostasis and gliotransmission.

## Zinc and astrocytes

Astrocytes have a variety of roles in regulating volume and composition of extracellular space, forming and controlling the blood-brain barrier (BBB) and also maintaining the architecture of gray matter (Kettenmann and Verkhratsky, 2008). Astrocytes are capable of rapidly accumulating Zn (Nolte et al., 2004) and they also express a range of ZnT’s including ZIP14 (Bishop et al., 2010) and ZnT3 (which is intimately involved in cognition) under stress conditions (Sun et al., 2012). It is known that glial cells in the cerebellum also express ZnT1, ZnT3, ZnT4 and ZnT6 (Wang et al., 2005). The biological implications, and indeed the cognitive effect, of this Zn accumulation and Zn transporter expression are currently unknown and remain an under-researched area. That being said, astrocytes take up glutamate from the synapse and convert it to glutamine which is subsequently released and retrieved by neurons for conversion back to glutamate and into the main inhibitory transmitter gamma-aminobutyric acid (GABA; Yeh et al., 2013). This glutamate-glutamine cycle is essential to maintaining glutamatergic and GABA-ergic neurotransmission, disruption of which could cause synaptic dysfunction (Yeh et al., 2013) and cognitive deficits. Moreover, activation of astrocytic G-protein coupled receptors stimulates the release of glutamate as well as potentiates NMDA receptor functions (Lee et al., 2007). Thus, the close association/co-release of Zn and glutamate at the synapse may, therefore, extend to a role for astrocytes/glia in the synaptic regulation of Zn that may be critical for the prevention of excitotoxicity or various other post-synaptic or cognitive processes.

Additionally, astrocytes increase intracellular Ca^2+^ levels in response to synaptic transmission (Morris et al., 2013). This may be important in LTP which is induced by high-frequency stimulation causing a sustained increase in transmission that usually depends on Ca^2+^ influx through NMDA receptors. A recent study by Han et al. (2013), which grafted human astrocytes into mice, showed enhanced LTP and improved performance on cognitive tests. It should be noted here that oxidative stress (previously mentioned as a major contributor to cellular damage) is known to affect metabolic pathways of astrocytes that are important for the delivery of metabolites to neurons (Theusen et al., 2010). This demonstrates a common cause of functional disruption across cell types essential to Zn homeostasis that may be especially detrimental at the synapse.

## Tripartite synapse

The role of Zn at synapses is particularly germane to both normal and pathological ageing. Neuroplasticity deteriorates in normal ageing and loss of synapses is a key indicator and currently the best correlate of cognitive decline in AD (Takahashi et al., 2010). However, the current study of synapses falls well-short of that necessary to fully comprehend the complex processes that occur. The model of a synapse over the past decades of research has been that of neuron-neuron electrical impulses, but recent advances in both scientific thinking and techniques has brought us to a more accurate representation. Currently the most accepted model is that of the “tripartite” synapse where the pre- and post-synaptic neuron terminals are physically enveloped by astrocytic processes which actively participate in synaptic neurotransmission. These astrocytic processes release a variety of neuroactive molecules including adenosine, GABA, prostaglandins and ATP which can influence both neuronal and synaptic physiology (Volterra and Bezzi, 2002).

Though the link has previously not been made, the evidence presented here suggests that there may be an interaction between neurons, astrocytes, Zn and glutamate that is critical to normal synaptic health and function, potentially affecting neurotransmission and other cellular cascades involved in cognition, as discussed earlier. This is an area of research that clearly needs further investigation, as the diminished learning and memory abilities seen in ageing may be caused by a failure in neuron-glia communications at the synapse that disrupts Zn homeostasis to result in downstream modifications to, among other things, synaptic ion channels.

## Microglia at the synapse

**Figure 2:**
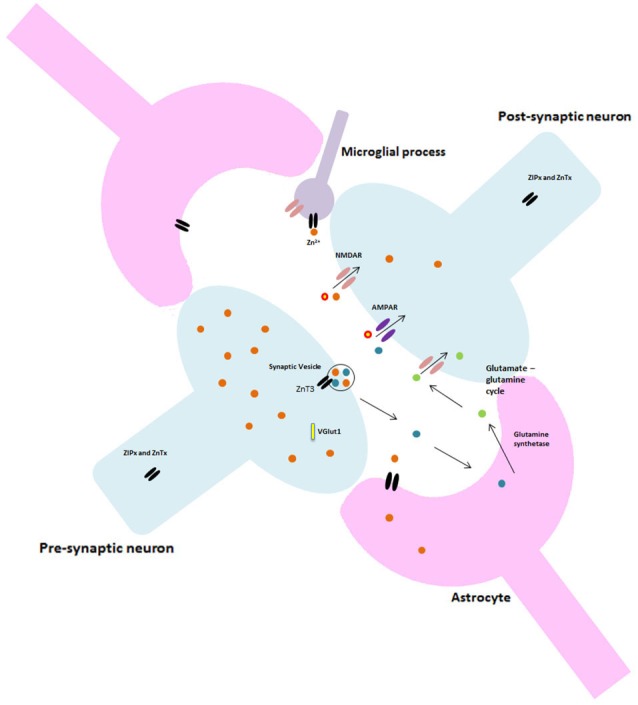
**Proposed glutamatergic synapse structure.** Including astrocytes and microglia.

In addition to the role of astrocytes at the synapse, recent studies investigating microglia suggest this tripartite system may be just as constraining to synaptic research as the previous model. Currently, the literature is dominated by what is thought to be the primary function of microglia; surveying the environment for damage and protecting neural material (Stollg and Jander, 1999). Resting microglia have small cell bodies and elongated processes, in response to a traumatic or pathogenic event they alter their morphology, withdraw their processes and become globular. These activated microglia are able to migrate to the damage site, phagocytose any cellular debris and release various neuroactive compounds (Wake et al., 2009). However, it is almost certain that microglia have a much larger role in normal brain function and cognition than previously thought. Resting microglia have been shown to be highly dynamic, extending and retracting processes with brief static periods, apparently at random (Nimmerjahn et al., 2005). Microglia express most of the major classes and subtypes of both excitatory and inhibitory neurotransmitter receptors and ion channels which have classically been found at neuronal synapses, though little is known about their role in inactivated microglia. Indeed, research by Wake et al. (2009) showed microglial processes making direct transient contact with synapses. Moreover, microglia appear to contact neighboring astrocytes, neuronal cell bodies and blood vessels (Morris et al., 2013). These findings have vast implications for our current understanding of both synaptic and cognitive functioning in that such findings require scientists to completely alter their perceptions and re-consider the current models of synapses as well as the dominant focus on neurons throughout past and present research and literature. Figure 2 illustrates the potential structure of this new synaptic model.

Microglia also release cytokines, which have central physiological roles in synaptic plasticity, neurogenesis and learning and memory in the normal brain (Morris et al., 2013) possibly through their influence on MT expression and hence Zn concentration (Vasto et al., 2008). Microglia may, however, have a more direct role in Zn homeostasis as Higashi et al. (2011) recently learned. Microglia can directly uptake Zn via ZIP1, which is also a trigger for sequential microglial activation. In a study by Knoch et al. (2008) the release of intraneuronal Zn^2+^ and a subsequent increase in neuronal voltage-gated K^+^ currents as caused by the release of ROS from activated microglia lead to neuronal cell death. This suggests the primary mechanism of neuronal apoptosis may in fact, be the earlier damage to glial cells. This is accordance with findings by Kaindl et al. (2008, 2012) that activation of microglial NMDA receptors results in an increase in oxidative stress *in vitro* (Kaindl et al., 2008, 2012). Glial senescence during ageing can also impact normal synapse function (Wong, 2013) and result in aberrant connectivity between neurons.

## Zinc, glia and pathological ageing

**Figure 3:**
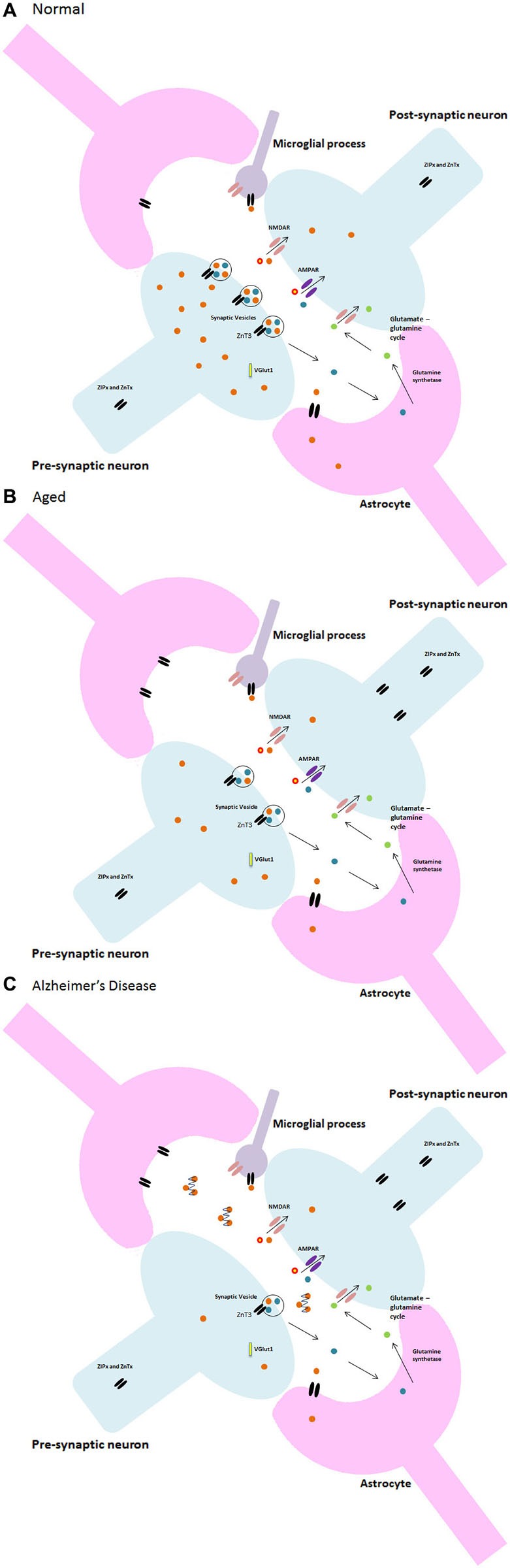
**Neuron-glia and zinc synaptic interactions in (A) normal, (B) aged, and (C) Alzheimer’s disease**.

Both Zn and glia appear to have multifarious roles within the brain, especially within synapses. Indeed, synaptic loss is the fundamental feature of the ageing brain that links neuropathology to cognitive decline in AD (Talantova et al., 2013). This is largely applicable to pathological ageing and neurodegenerative disorders such as AD wherein the abnormal deposition of misfolded Aβ peptide into plaques, which bind Zn, results in a significant decrease in intracellular Zn (Bush et al., 1994). Moreover, the plaques are in abundance in brain areas with high densities of glutamatergic synapses such as the hippocampus, exhibiting a similar distribution to that of Zn with glutamatergic neurons. Additionally, microglia have been suggested to play a role in plaque formation (Stollg and Jander, 1999). Further research by Desphande et al. (2009) clearly demonstrated oligomers of Aβ interfering with synaptic function, suggesting that Zn at the NMDA receptor attracts the Aβ in addition to its high binding affinity to synapses. Keeping the aforementioned information in mind, it is reasonable to suggest that AD may be the result of synaptic dysfunction caused by a disruption of the fine and complex interplay between Zn, neuronal, glial and microglial communication that occurs within the synapse. Due to the high binding affinity of Aβ to both Zn and synapses, upon activation of the pre-synaptic neuron and the subsequent release of Zn into the synapse, the Zn can be captured by the Aβ and lodged within the plaque to ultimately disrupt synaptic transmission. A decrease in available Zn for neurotransmission and calcium signaling then causes downstream errors that may result in further Zn dyshomeostasis in a negative feedback loop eventually leading to glial damage and apoptosis through microglial activation. Disruption of cytokine signaling and failure of the signaling mechanisms maintaining the phenotype of microglia in the normal brain may contribute to learning and memory dysfunction and synaptic pathologies such as AD or dementia which, in some of its forms, is at its onset a result of a failure to maintain microglia in their ramified state (Morris et al., 2013). A diagrammatic representation of the change in Zn^2+^ in the progression from normal to pathological ageing is illustrated in Figure 3.

## Conclusion

Herein evidence supporting a link between Zn, glia and cognitive decline has been presented and discussed. The research thus far suggests the possibility of a feedback loop between Zn homeostasis, synaptic excitation, neurons, astrocytes and microglia. Perhaps the most appropriate definition is that of Morris et al. (2013) that a synapse is “a complex, dynamic and often transient structure involving several cells interacting with a sophisticated extracellular matrix and milieu”. The contribution of microglia and astrocytes to synaptic plasticity mechanisms relevant to learning and memory must be included in studies. Only by including these cell types in future research will we come to truly understand the intricate molecular mechanisms underlying the ageing processes; thereby discovering potential avenues for intervention to ensure that we are able to enjoy our twilight years to the best of our cognitive ability.

## Conflict of interest statement

The authors declare that the research was conducted in the absence of any commercial or financial relationships that could be construed as a potential conflict of interest.
